# Exploring the prognostic role of microbial and genetic markers in lung squamous cell carcinoma

**DOI:** 10.1038/s41598-025-88120-2

**Published:** 2025-02-06

**Authors:** Fan Yang, Xiaodong Jia, Zihuan Ma, Siyao Liu, Chunzi Liu, Dan Chen, Xiuju Wang, Niansong Qian, Hui Ma

**Affiliations:** 1 Senior Department of Oncology, The Fifth Medical Center of Chinese PLA General Hospital, Beijing, China; 2 Beijing ChosenMed Clinical Laboratory Co. Ltd, Jinghai Industrial Park, Economic and Technological Development Area, Beijing, 100176 China; 3 Senior Department of Thoracic Oncology, Respiratory and Critical Care Medicine, The Eighth Medical Center of People’s Liberation Army General Hospital, Beijing, China; 4 Nursing Department of Chinese, PLA General Hospital, Beijing, China

**Keywords:** Lung squamous cell carcinoma, Microbiome, Prognosis, Risk score model, Cancer, Cancer models

## Abstract

Despite advances in diagnostic and therapeutic strategies, the prognosis of lung squamous cell carcinoma (LUSC) patients remains poor, and the potential of microbiome-based prognostic biomarkers and therapeutic targets remains largely unexplored. LUSC patient data from The Cancer Genome Atlas (TCGA), including microbial genus level abundance data and RNA sequencing (RNA-Seq) data, were used as a training dataset. Two other independent datasets GSE19188 and GSE157009 serve as validation datasets. A microbiome-based risk score (RS) model was constructed by univariate Cox regression analysis combined with the least absolute contraction and selection operator (LASSO) regression. 18 microbial genera were found to be significantly associated with RFS in LUSC patients. The microbial signature built with these microbial genera, exhibited robust predictive accuracy in both the training and validation datasets. Furthermore, hub mRNA between high- and low-risk groups were selected by XGBOOST and intersect with mRNAs screened by univariate Cox regression analysis, finally identifying four mRNA significantly associated with LUSC prognosis. This study reveals a complex interplay between the lung microbiome and genetic biomarkers, and identifies specific microbial-based and mRNA associated with prognosis in LUSC. These findings provide a basis for future studies aimed to elucidate the mechanisms underlying these associations and provide potential biomarkers for guiding treatment decisions and improving patient outcomes.

**Supplementary Information:**

The online version contains supplementary material available at 10.1038/s41598-025-88120-2.

## Introduction

Lung squamous cell carcinoma (LUSC) is a predominant histological subtype of non-small cell lung cancer (NSCLC), representing a significant proportion of lung cancer cases worldwide. Even with progress in diagnostic and treatment methods, LUSC patients still face poor outcomes, with a five-year survival rate under 20% and a 33% chance of local recurrence within 2 years^1^. This underscores the urgent need for novel prognostic biomarkers and therapeutic targets to improve patient outcomes^2^.

Recent studies have highlighted the critical impact of the tumor microenvironment, including microbial communities, on cancer initiation and progression. The human microbiome, particularly the gut microbiome, has been found to affect systemic inflammation, immune responses, and even the efficacy of cancer therapies. Microbial dysbiosis, an imbalance in microbial communities, has been implicated in various malignancies, including colorectal, gastric, and liver cancers^3^. These findings suggest that the microbiome may modulate tumor behavior through multiple mechanisms, including modulation of the immune system, production of microbial metabolites, and alteration of the tumor microenvironment^4^. In the context of lung cancer, the lung microbiome is of particular interest. The lung, once considered a sterile environment, is now known to harbor a diverse microbial community. Variations in the lung microbiome have been connected to chronic respiratory conditions and might influence the pathogenesis of lung cancer^5^. Specific microbial taxa have been linked to inflammatory pathways that could contribute to tumor initiation and progression. For instance, specific bacteria can trigger chronic inflammation, a recognized risk factor for cancer. Moreover, microbial metabolites like short-chain fatty acids and secondary bile acids can affect cellular proliferation, apoptosis, and DNA repair mechanisms^6–8^. Despite the growing recognition of the microbiome’s role in cancer, the specific impact of microbial communities within the lung on LUSC prognosis has not been thoroughly investigated. Given the intricate interplay between the microbiome and the host immune system, understanding the microbial composition associated with LUSC could provide valuable insights into the mechanisms driving cancer progression and identify potential biomarkers for prognosis and therapeutic targets. A study demonstrates a notable reduction in certain bacterial groups in lung cancer tissues, indicating a link between lung microbiome imbalances and lung cancer. This suggests further investigation is necessary to clarify these microbial alterations’ impact on cancer^7^. Liu’s study highlights the significant role of microbiome imbalances in lung cancer progression. It discusses dysbiosis can promote tumor development and examines potential therapeutic strategies targeting the microbiome to treat lung cancer^9^. Another study reveals that lung microbiome imbalances contribute to lung cancer by promoting tumor growth and modulating immune responses, highlighting the therapeutic potential of targeting the microbiome to improve cancer treatment outcomes^10^.

In addition to microbial factors, the molecular characterization of tumors through high-throughput sequencing technologies has provided valuable insights into the genetic and transcriptomic alterations associated with cancer prognosis. RNA sequencing (RNA-Seq) data have been extensively utilized to identify differentially expressed genes (DEGs) and construct prognostic models based on gene expression profiles. Integrating these molecular data with microbial abundance data could potentially unveil novel prognostic markers and improve the predictive accuracy of existing models^11^.

**Fig. 1:**
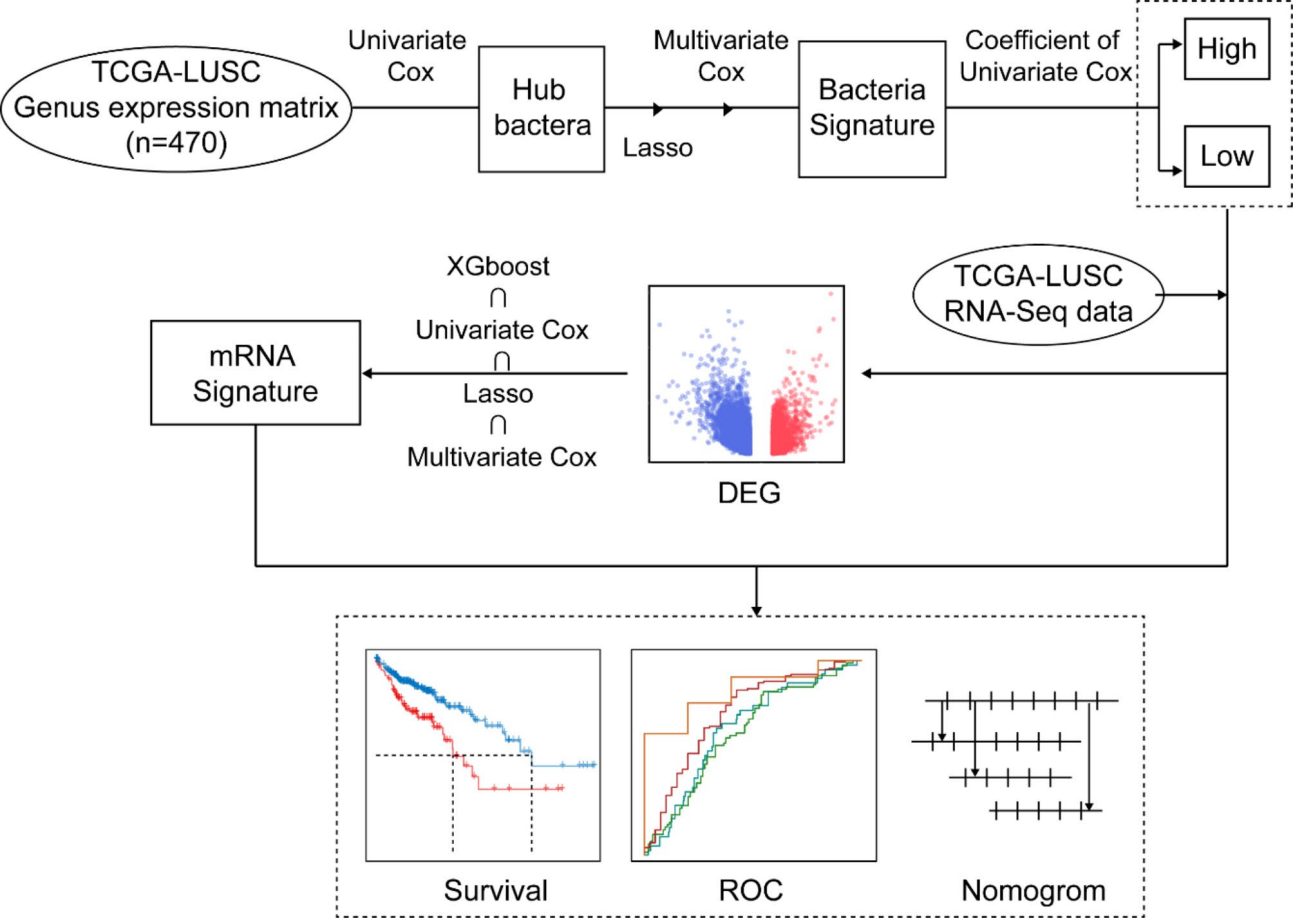
The workflow of this study.

This study aims to identify prognostic microbial markers in LUSC by comprehensively analyzing microbial genus-level abundance and RNA-Seq data. We hypothesize that specific microbial genera are significantly associated with LUSC prognosis and that integrating these microbial markers with mRNA expression data can enhance the prognostic accuracy of existing models (Fig. 1).

## Methods

Figure 1 illustrates the method of present research

### Sample collection and data acquisition

The microbial genus-level abundance data for this study were obtained from the publicly available dataset published by Wei et al.^12^. These data were derived from whole-genome sequencing (WGS) data in The Cancer Genome Atlas (TCGA) and processed using rigorous quality control measures, including contaminant removal, alignment to microbial reference databases, and in silico decontamination protocols. Details of the data generation process are described in the original study. Additionally, two independent datasets, GSE19188 and GSE157009, were sourced from the Gene Expression Omnibus (GEO) database. These datasets served as validation datasets to assess the stability and reproducibility of our constructed models.

### Analysis of microbial genus -level abundance data

We initially conducted a univariate Cox proportional hazards analysis on the microbial genus-level abundance data from our training cohort to pinpoint microbial genera linked to the prognosis of LUSC patients. Subsequently, we applied LASSO (Least Absolute Shrinkage and Selection Operator) to further refine those microbial genera with the most significant prognostic impact. To enhance the reliability and precision of the model, we determined the best regularization parameter for LASSO regression using 10-fold cross-validation. This process identified key microbial genera associated with patient survival.

### Multivariate cox regression analysis and risk score model construction

Using the selected microbial genera from the LASSO regression, we conducted multivariate Cox regression analysis to determine the final core microbial composition. The risk score for each patient was calculated using the formula:

$$\:Risk\:Score={\sum\:}_{i=i}^{n}(\beta\:i\text{}\times\:Xi\text{})$$

where *β*_*i*_ represents the regression coefficient from the multivariate Cox regression analysis, and *X*_*i*_ represents the abundance value of the corresponding microbial genus. Patients were then classified into high-risk and low-risk groups based on the median risk score.

### Prognostic performance evaluation

To evaluate the prognostic performance of the risk score model, Kaplan-Meier (KM) survival curves were plotted, and the Log-rank test was used to compare survival differences between high-risk and low-risk groups. Additionally, Receiver Operating Characteristic (ROC) curves and the Area Under the Curve (AUC) were used to further assess the model’s predictive performance.

A nomogram was constructed to provide a visual representation of the predictive model, integrating multiple prognostic factors to estimate the survival probability for individual patients. The performance and calibration of the nomogram were assessed using calibration curves and decision curve analysis (DCA).

### Differential gene analysis

RNA-Seq data were analyzed using XGBOOST (Extreme Gradient Boosting) to identify hub mRNAs between high-risk and low-risk groups. These hub mRNAs were then intersected with significant mRNAs identified in the univariate Cox regression analysis to identify potential key genes. This intersection aimed to pinpoint genes with crucial roles in the prognosis of LUSC.

### mRNA predictive model construction and validation

LASSO regression analysis was performed on the identified key mRNAs to select the optimal mRNA combination, followed by multivariate Cox regression analysis to construct a predictive model consisting of four mRNAs. The risk score for this model was calculated using the formula:

$$\:mRNA\:Risk\:Score={\sum\:}_{j=i}^{n}(\beta\:j\text{}\times\:Yj\text{})$$

where *β*_*j*_ represents the regression coefficient from the multivariate Cox regression analysis, and *Y*_*j*_ represents the expression level of the corresponding mRNA.

The model’s robustness and generalizability were validated using the external datasets GSE19188 and GSE157009. KM survival curves and ROC curves were employed to assess the model’s performance in these datasets.

A nomogram was also constructed based on the mRNA predictive model to provide a visual and quantitative method for clinicians to predict the survival probability of LUSC patients. The nomogram’s performance was evaluated using calibration plots and the C-index.

### Mutation landscape analysis

The mutation landscape of the mRNA predictive model was analyzed using mutation data from the TCGA database. The mutation frequency and spectrum between high-risk and low-risk groups were compared, and the tumor mutation burden (TMB) for each group was calculated. The Maftools package was used to visualize the mutation spectrum.

### Immune microenvironment analysis

To investigate differences in the immune microenvironment between high-risk and low-risk groups, the CIBERSORT algorithm was used to analyze the relative abundance of 22 immune cell types in each sample. The Mann-Whitney U test was employed to compare the immune cell abundance between the high-risk and low-risk groups.

### TCGA clinical stratification analysis

To evaluate the applicability and stability of the mRNA predictive model across different clinical characteristics, we conducted multidimensional stratified analysis using clinical information from the TCGA database. Patients were grouped by clinical stage (Stage I, II, III, IV), and KM survival analysis and Log-rank tests were performed within stage I&II and stage III&IV group to compare survival between high-risk and low-risk groups. ROC curves and AUC values were also calculated for each stage group. Patients were divided into two groups based on age (< 68 years and ≥ 68 years), and KM survival analysis was conducted within each age group. ROC curves and AUC values were also calculated for each age group. Patients were grouped by gender (male and female), and KM survival analysis was performed within each gender group. ROC curves and AUC values were also calculated for each gender group. Patients were divided based on smoking history (smokers and non-smokers), and KM survival analysis was conducted within each group. ROC curves and AUC values were also calculated for each group.

### Drug sensitivity analysis

To explore the association between the predictive model and drug sensitivity, the half-maximal inhibitory concentration (IC50) values for various chemotherapeutic agents were obtained from the Genomics of Drug Sensitivity in Cancer (GDSC) database. The correlation between the risk scores derived from our model and the IC50 values of different drugs was assessed. The Wilcoxon rank-sum test was used to compare the drug sensitivity between high-risk and low-risk groups.

### Statistical analysis

All statistical analyses were conducted using R software (version 4.0.3). Key R packages included “survival,” “glmnet,” “xgboost,” “maftools,” and “pROC.” All statistical tests were two-sided, with a P-value < 0.05 considered statistically significant.

## Results

### Screening of microbial genus-level abundance data

**Fig. 2:**
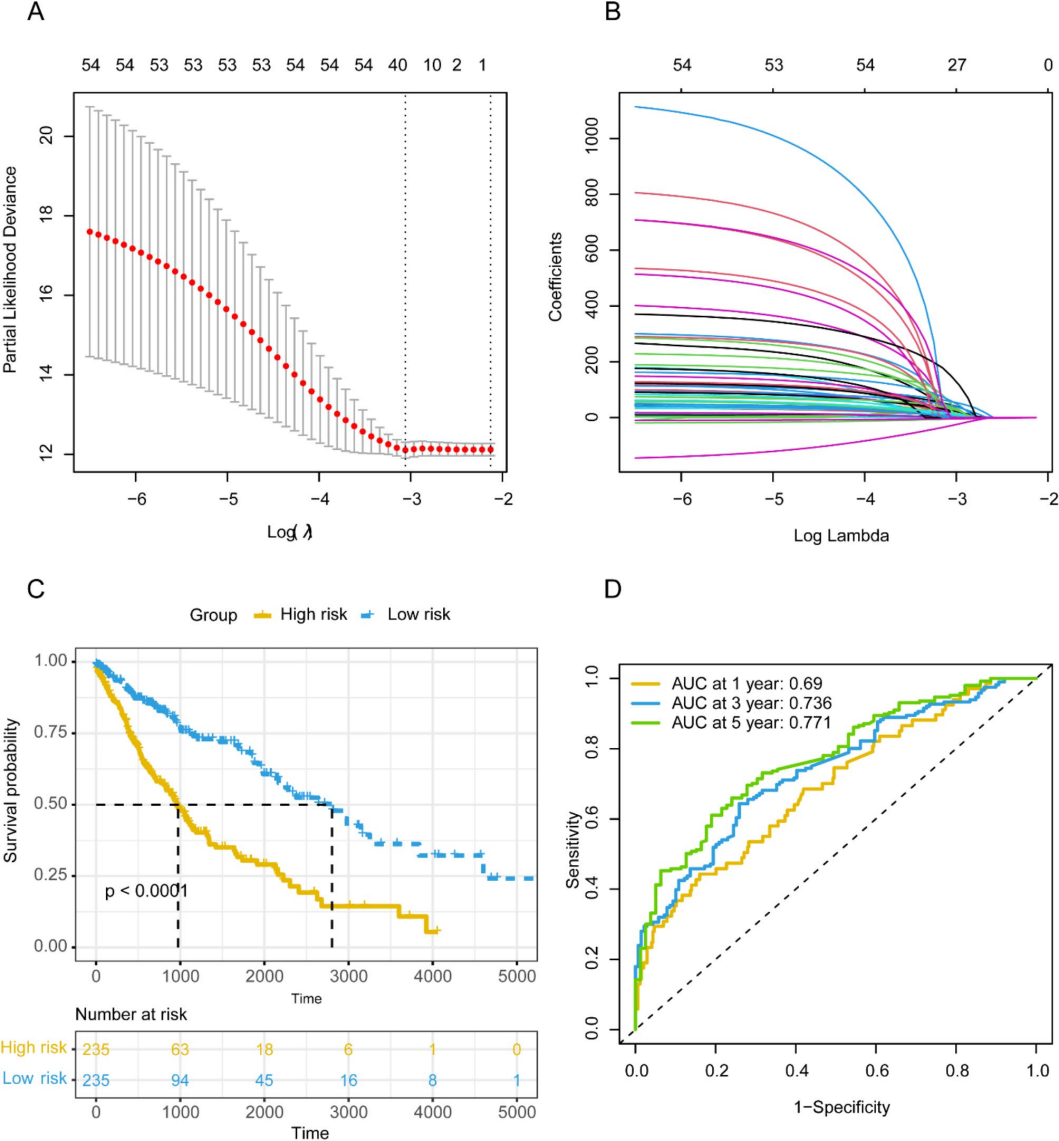
Establishment of microbial prognostic signatures. (**A**) The process of selecting the optimal value of the parameter λ. (**B**) LASSO coefficient profiles of the variables. (**C**) The K-M plot of the two clusters. (**D**) The ROC curves of the microbial prognostic signatures.

In univariate Cox regression analysis, 55 microorganisms were significantly associated with survival outcomes in patients with squamous lung cancer (*P* < 0.05, Table S1). A total of 55 microorganisms of the microbial dataset were subjected to LASSO analysis and 27 microorganisms were obtained, and the results of the LASSO analysis are shown in Fig. 2A and B. These 27 microorganisms were then subjected to multivariate Cox regression analysis, which yielded 18 microorganisms (Azotobacter, Buchnera, Corynebacterium, Delftia, Desmospora, Dinoroseobacter, Dokdonia, Jonesia, Acetobacterium, Leptolyngbya, Methylibium, Mycoplasma, Nocardioides, Nocardiopsis, Pasteurella, Proteus, Yersinia and Lautropia) that were significantly associated with survival outcomes in patients with squamous lung cancer (*P* < 0.05, Table S2).

### Microbiology prognostic risk score signature

The microbiology RS for each patient was calculated using the following formula: RS = (Exp_*Azotobacter*_×2.87276530576684) + (Exp_*Buchnera*_×64.2217461372653) + (Exp_*Corynebacterium*_×(-9.65619513527936)) + (Exp_*Delftia*_×64.870930443892) + (Exp_*Desmospora*_×84.1763042921373) + (Exp_*Dinoroseobacter*_×209.920581156474) + (Exp_*Dokdonia*_×110.477522016722) + (Exp_*Jonesia*_×86.3478620425963) + (Exp_*Acetobacterium*_×191.032983731884) + (Exp_*Leptolyngbya*_×(-10.2237192718484)) + (Exp_*Methylibium*_×41.857687812083) + (Exp_*Mycoplasma*_×(-121.362117965697)) + (Exp_*Nocardioides*_×57.7465602770095) + (Exp_*Nocardiopsis*_×22.2910524592335) + (Exp_*Pasteurella*_×150.610836388783) + (Exp_*Proteus*_×269.523366330274) + (Exp_*Yersinia*_×50.2255262650574) + (Exp_*Lautropia*_×36.784435452829). After calculating the microbial risk score for each patient in the training dataset, patients were categorized into high- and low-risk groups based on the median risk score, and Figure S1 shows the distribution of risk scores (Figure S1A), survival profiles (Figure S1B), and expression level of the 18 microorganisms that comprise the microbial prognostic signature of patients in the training dataset (Figure S1C). Later we used the KM survival analysis to show the prognostic differences between the high and low risk groups. As can be seen in Fig. 2C, there was a significant difference (*p* < 0.0001) was observed in the OS between the different risk groups. In the ROC curve, the microbial prognostic model demonstrated good predictive ability in patients’ 1-year, 3-year, and 5-year survival (AUC_1-year_ = 0.690, AUC_3-year_ = 0.736, AUC_5-year_ = 0.771, Fig. 2D).

### Evaluation of the predictive performance of the microbiology prognostic risk score signature and immune microenvironment

**Fig. 3:**
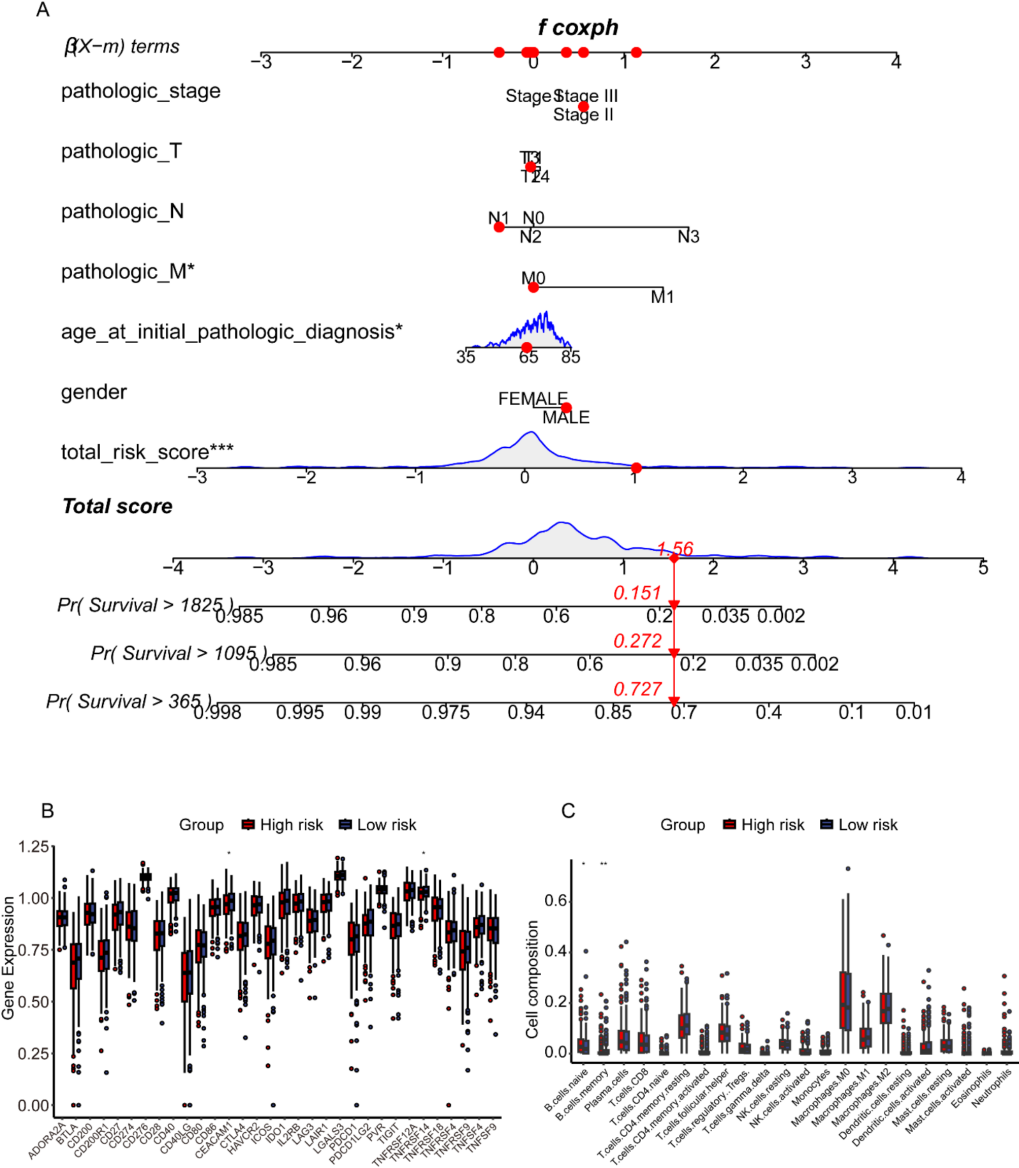
Assessment of microbiological prognostic signatures. (**A**) Nomogram used to compare microbial prognostic signatures with other clinical indicators. (**B**). The expression of immune checkpoint-related genes in the high- and low-risk of patients in the microbial dataset. (**C**) the immune cell infiltration in the high- and low-risk of patients in the microbial dataset.

In the established nomogram, we compared the microbial prognostic signature to the clinical indicators of gender, age, pathologic M, pathologic N, pathologic T, and pathologic stage. As can be seen in Fig. 3A, the risk score of the microbial prognostic model was weighted much more than the other clinical indicators in the nomogram while being significant. As can be seen in Figure S1D, the net benefit of the microbial signature is much higher than the remaining clinical indicators. And as can be seen in the calibration curves (Figure S1E), the microbial prognostic signature predicted different timepoint survival in patients with squamous lung cancer in a high degree of agreement with actual survival. As seen in the immune microenvironment analysis, both immune checkpoints (Fig. 3B), *CEACAM1* and *TNFRSF14*, were expressed at significantly higher levels in low-risk patients than in high-risk patients. Naive and memory B cells showed significant differences between two risk groups (Fig. 3C).

### Screening of mRNA data

**Fig. 4:**
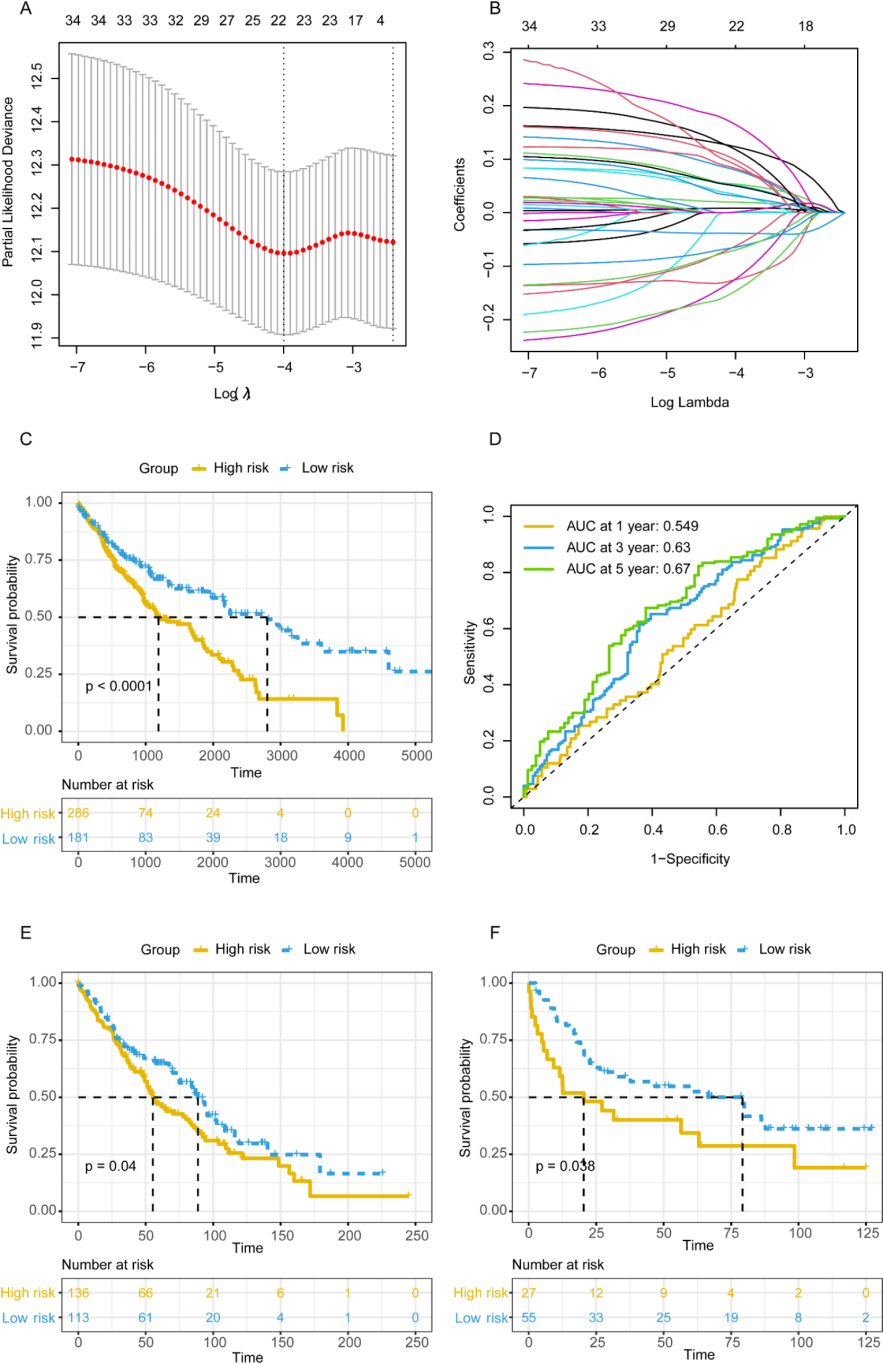
Establishment of mRNA prognostic signatures. (**A**) The process of selecting the optimal value of the parameter λ. (**B**) LASSO coefficient profiles of the variables. (**C**) The K-M plot of the two clusters in the training dataset. (**D**) The ROC curves of the mRNA prognostic signatures in the training dataset. (**E**) The K-M plot of the two risk group in GSE157009 dataset. (**F**) The K-M plot of the two risk group in GSE19188dataset.

By employing XGBOOST to screen for feature genes associated with high and low risk groups in the microbial model, a total of 831 mRNAs were identified,, as shown in Table S3, after which 33 mRNAs were obtained by taking the intersection with 904 mRNAs screened by the univariate COX analysis (Table S4) that were significantly correlated with the prognosis of the patients. After that, LASSO analysis (Fig. 4A and B) was used to further screen the mRNAs and 23 mRNAs were remaining. After multivarate COX analysis (Table S5), finally 4 mRNAs were screened which were highly correlated with the prognosis of patients with squamous lung cancer.

### mRNA prognostic risk score signature

The mRNA RS for each patient was calculated using the following formula: RS = (Exp_*C9orf131*_ × 0.194883323292564) + (Exp_*SLN*_×0.103580235916565) + (Exp_*SSX5*_ × 0.139813921970907) + (Exp_*FAM110A*_×0.19531765239501). After calculating the microbial risk score for each patient in the training dataset, patients were categorized into high- and low-risk groups based on the cut off value (cut off = 2.197), and Figure S2 shows the distribution of risk scores (Figure S2A), survival profiles (Figure S2B), and expression level of the 18 genes that comprise the mRNA prognostic signature of patients in the training dataset (Figure S2C). The significant difference (*p* < 0.0001) in OS was oberserved among the distinct risk groups (Fig. 4C). In the ROC curve, the microbial prognostic model demonstrated good predictive ability in patients’ 1-year, 3-year, and 5-year survival (AUC_1-year_ = 0.690, AUC_3-year_ = 0.736, AUC_5-year_ = 0.771, Fig. 4D).

### Evaluation of the predictive performance of the mRNA prognostic risk score signature and immune microenvironment

**Fig. 5:**
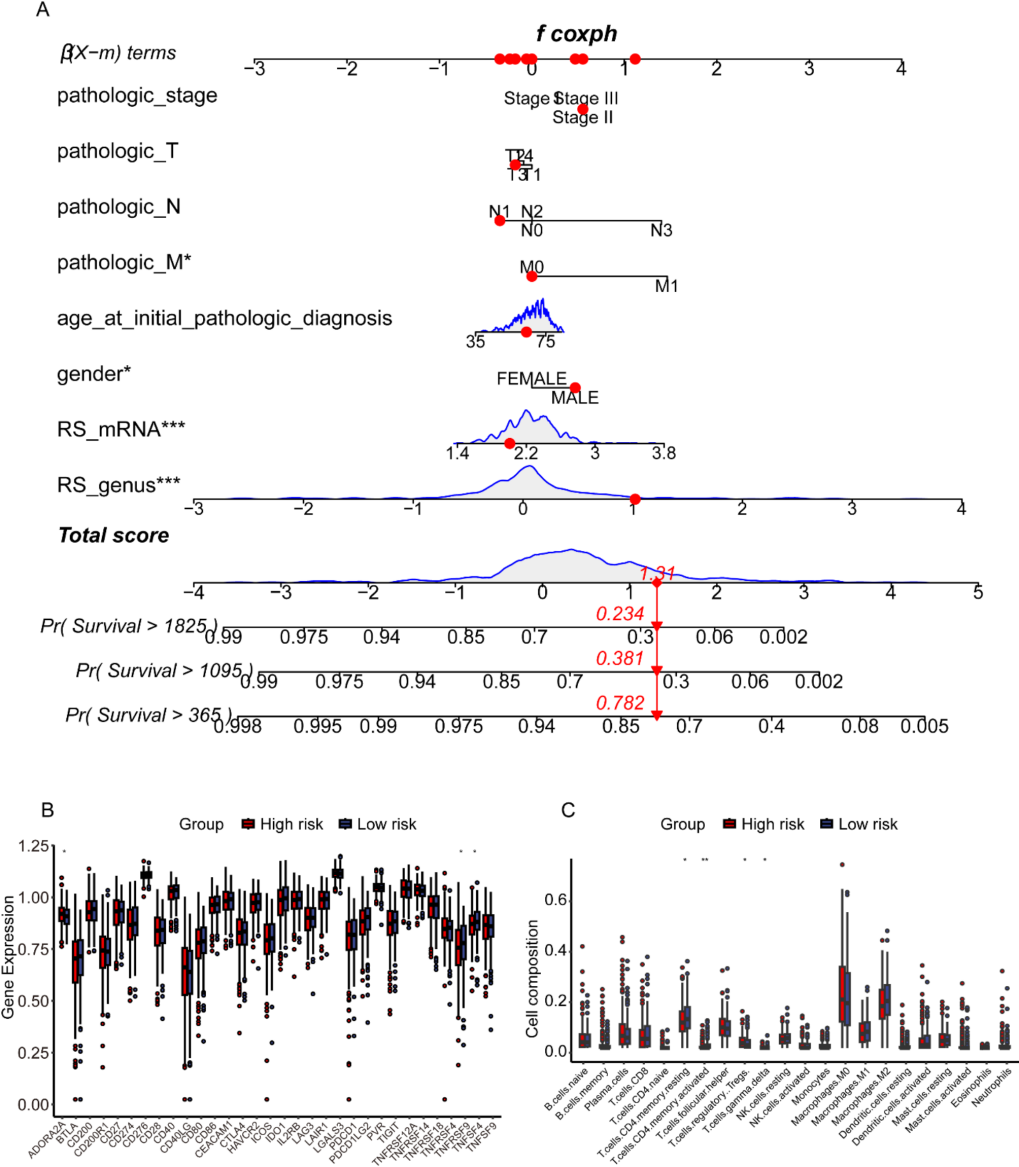
Assessment of mRNA prognostic signatures. (**A**) Nomogram used to compare microbial and mRNA prognostic signatures with other clinical indicators. (**B**) The expression of immune checkpoint-related genes in the high- and low-risk of patients in the training set. (**C**) the immune cell infiltration in the high- and low-risk of patients in the training set.

**Fig. 6:**
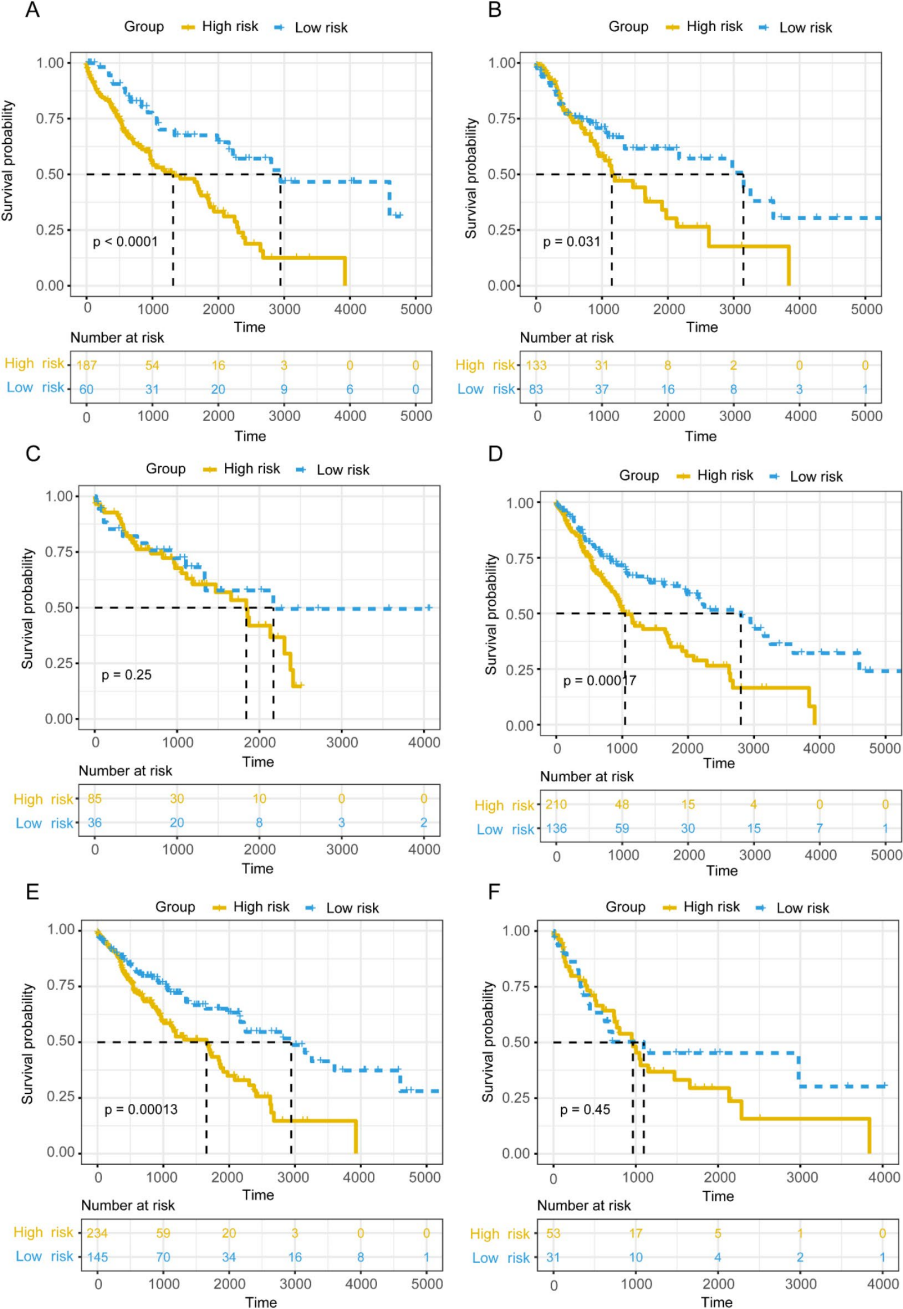
Validation of mRNA prognostic signatures in different patient stratifications. (**A**) The K-M plot of patients with age greater than 68 in the training dataset. (**B**) The K-M plot of patients with age less than 68 in the training dataset. (**C**) The K-M plot of female patients in the training dataset. (**D**) The K-M plot of male patients in the training dataset. (**E**) The K-M plot of stage I and II patients in the training dataset. (**F**) The K-M plot of stage III and IV patients in the training dataset.

The ability of the mRNA prognostic signature to predict the prognostic status of patients with squamous lung cancer was subsequently validated in two datasets, GSE19188 and GSE157009. Respectively, patients were categorized into two risk group with mRNA prognostic signature threshold at 0.0562 and 3.342, and significant survival differences were observed between these two groups of patients as seen in Fig. 4E and F. In the established nomogram, we compared the mRNA and microbial prognostic signature to the clinical indicators of gender, age, pathologic TNM stage. As can be seen in Fig. 5A, the risk score of the mRNA and microbial prognostic signature was weighted much more than the other clinical indicators in the nomogram while being significant. As can be seen in Figure S2D, the net benefit of the mRNA prognostic signature risk score is lower than the microbial prognostic signature. As can be seen in the calibration curves (Figure S2E), the mRNA prognostic signatures predicted 1-, 3- and 5-year survival in patients with squamous lung cancer in a high degree of agreement with actual survival. As seen in the immune microenvironment analysis, both immune checkpoints (Fig. 5B), *ADORA2A*, *TNFRSF9* and *TNFSF4*, were expressed at significantly higher levels in low-risk patients than in high-risk patients. In immune infiltration it was seen that T cells CD4 memory resting, T cells CD4 memory activated, T cells regulatory tregs and T cells gamma delta were significantly different between high and low risk groups (Fig. 5C). Later, to further validate the predictive ability of the mRNA prognostic model, we validated it in different patient stratifications. As can be seen in Fig. 6A and B, the mRNA prognostic model can be effectively categorized into high and low risk groups in both those greater than 68 years of age as well as those less than 68 years of age (*p* = 0.031 and *p* < 0.0001). Also, the mRNA prognostic signature was unable to categorize female patients into high and low risk groups of patients with significant prognostic differences (*p* = 0.25, Fig. 6C), but this could be done in male patients (*p* = 0.00017, Fig. 6D). Similarly, the mRNA prognostic signature was effective in predicting patient prognosis in stage I and II patients (*p* = 0.00013, Fig. 6E), but could not do so in stage III and IV patients (*p* = 0.45, Fig. 6F).

## Discussion

The study’s findings provide critical insights into the role of specific microbial genera and genetic markers in the prognosis of lung squamous cell carcinoma (LUSC). These discoveries are significant not only for their potential as biomarkers but also for what they reveal about the underlying biological mechanisms at play.

The 18 microbial genera identified, including *Azotobacter*, *Buchnera*, *Corynebacterium*, *Delftia*, and *Mycoplasma*, among others, show a significant association with patient survival outcomes (Fig. 2C and D). This suggests that these microbes may contribute to or influence the tumor microenvironment. The presence of these specific microbes could modulate immune responses, potentially affecting the inflammatory milieu within the lungs^13^. Chronic inflammation is well-established as a promoter of cancer progression^14^; hence, the differential abundance of these microbes might reflect or induce variations in inflammatory pathways, thereby influencing tumor behavior^15,16^.

The concept of microbial dysbiosis—an imbalance in microbial populations—being linked to cancer progression is not new, but its specific application to lung cancer is still emerging^17–19^. This study provides evidence that lung microbiota may not merely be passive inhabitants but active players in the carcinogenic process (Fig. 2C and D). This aligns with findings in other cancer types where the microbiome can alter the efficacy of therapies or directly interact with tumor cells^16^. A number of studies concentrated on the microbes in digestive tract, where the most abundant microorganisms are colonized, and found that specific bacteria can modulate the host’s immune system, either enhancing or inhibiting tumor progression depending on the context^16,17,20–24^. However, since the lung microbiome directly contribute to the microenvironment in which the lung tumor developed, the role of microbes in lung is of particular importance to be investigated.

In addition to the microbial aspect, the study also identified four mRNAs—*C9orf131*.

, *SLN*, *SSX5*, and *FAM110A*—as significantly correlated with LUSC prognosis. These mRNAs likely play diverse roles in cellular processes pivotal to cancer biology. *SLN* is primarily known for its role in regulating calcium ion transport in muscle tissue^25^, but recent studies have suggested it might also be involved in cellular signaling pathways pertinent to cancer^25^. *SSX5* belongs to the *SSX* gene family, which has been studied for its role in various cancers, including synovial sarcoma. The expression of *SSX* proteins in cancer cells often correlates with more aggressive tumor behavior, suggesting a potential role in cell proliferation or immune evasion^26–28^. A previous study found that *FAM110A* was expressed at significantly higher levels in tumor samples in LUSC than in control samples and was significantly correlated with the prognosis of LUSC patients^29^. *FAM110A* has been associated with cell cycle regulation, and its altered expression could affect cellular proliferation and apoptosis, critical processes in cancer development and progression^30^.

The combined analysis of these mRNAs with microbial data enhances the understanding of the tumor microenvironment in LUSC. The interplay between genetic factors and microbial presence could offer insights into the mechanisms by which the tumor microenvironment influences cancer progression. For instance, the identified mRNAs may interact with microbial metabolites or influence the immune response, thereby modulating the tumor’s behavior and the patient’s response to treatment^31,32^.

This study’s findings have important implications for clinical practice. By providing a more nuanced understanding of the factors influencing LUSC prognosis, these results could inform the development of more targeted therapies. The identified microbial and mRNA markers could serve as the basis for diagnostic tests that help stratify patients by risk, allowing for more personalized treatment strategies. Moreover, the findings underscore the potential for integrating microbiome analysis into routine clinical practice, not only for prognosis but also for monitoring the effectiveness of treatment and potentially modulating the microbiome to improve therapeutic outcomes^33,34^.

In conclusion, this study advances our understanding of LUSC by highlighting the complex interplay between the lung microbiome and host genetic factors. The identification of specific microbial and mRNA markers associated with patient prognosis provides a foundation for future research aimed at elucidating the detailed mechanisms underlying these associations. Further studies are needed to validate these findings and explore the potential for these biomarkers in guiding treatment decisions and improving patient outcomes. The integration of microbiome and transcriptomic data represents a promising frontier in cancer research, offering the potential for novel therapeutic targets and more personalized approaches to cancer care.

## Electronic supplementary material

Below is the link to the electronic supplementary material.

Supplementary Material 1

Supplementary Material 2

Supplementary Material 3

Supplementary Material 4

Supplementary Material 5

Supplementary Material 6

Supplementary Material 7

Supplementary Material 8

## Data Availability

All data generated or analyzed during this study are included in this published article.
