# Supplementary figures and images for: Exploring the prognostic role of microbial and genetic markers in lung squamous cell carcinoma
Source: Sci Rep. 2025 Feb 6;15:4499. doi: 10.1038/s41598-025-88120-2 (PMC11802751; doi:10.1038/s41598-025-88120-2)

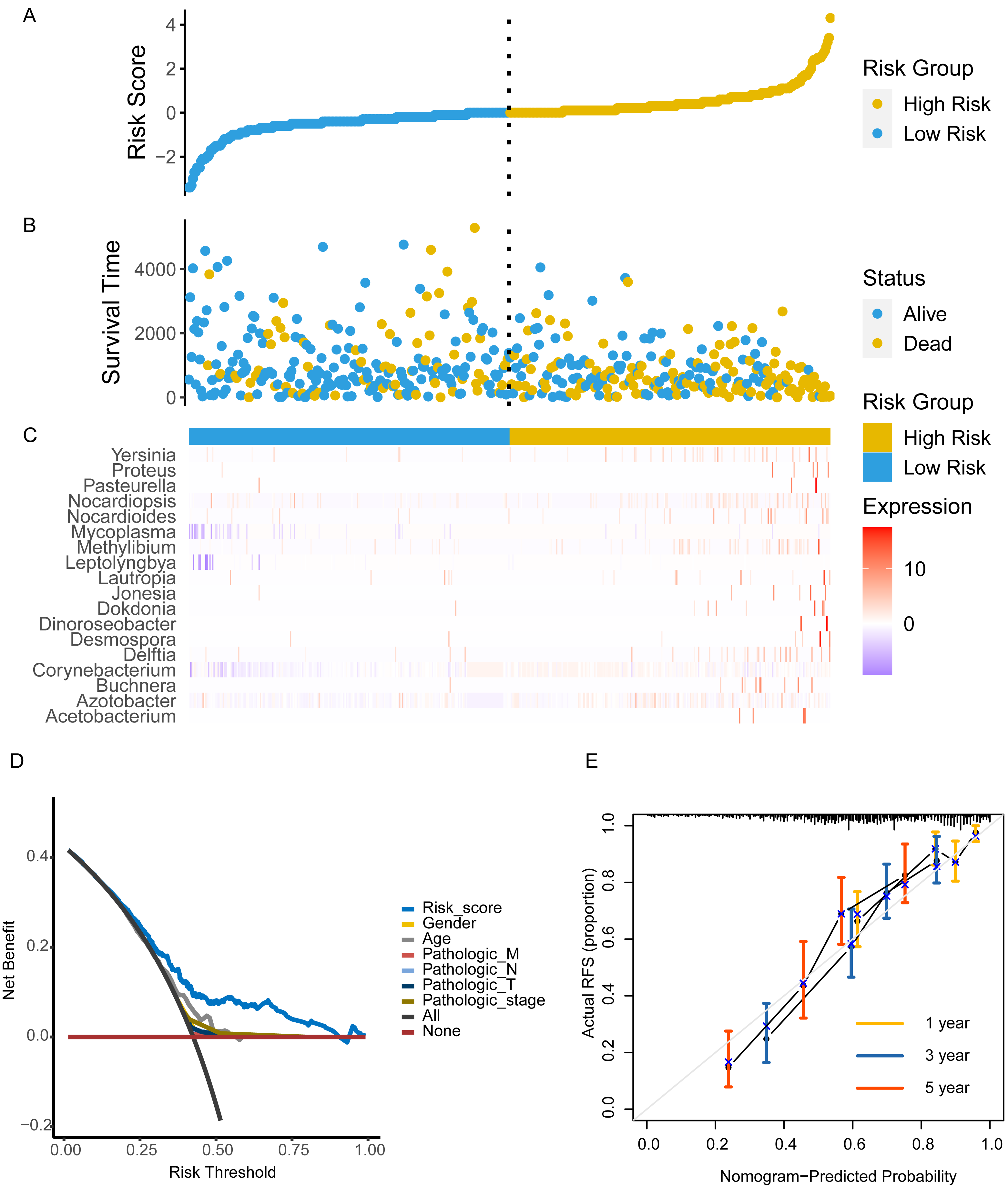

Supplement: Supplementary file 1 — Supplementary Material 1 [file 41598_2025_88120_MOESM1_ESM.tif]

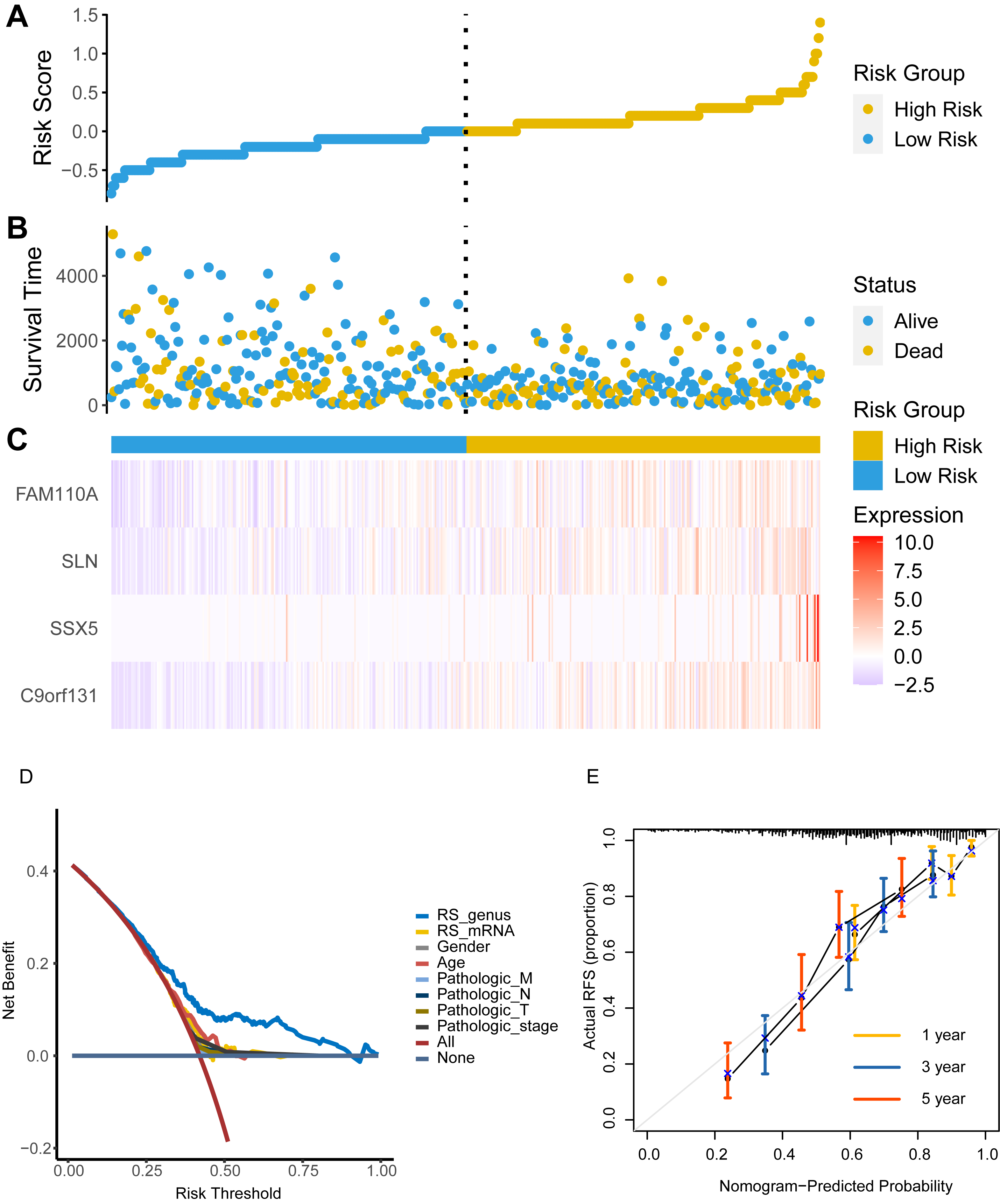

Supplement: Supplementary file 2 — Supplementary Material 2 [file 41598_2025_88120_MOESM2_ESM.tif]
