# Supplementary material for: Exploring the prognostic role of microbial and genetic markers in lung squamous cell carcinoma
Source: Sci Rep. 2025 Feb 6;15:4499. doi: 10.1038/s41598-025-88120-2 (PMC11802751; doi:10.1038/s41598-025-88120-2)
Supplement: Supplementary file 3 — Supplementary Material 3 [file 41598_2025_88120_MOESM3_ESM.docx]

**Figure S1**: Grouping of the microbial dataset. (**A**) Distribution of risk scores for patients in the high and low risk groups in the microbial dataset. (**B**) The heatmap of the expression of 18 microbial genera. (**C**) Distribution of survival for patients in the high and low risk groups. (**D**) The net benefit of the indicators. (**E**) Calibration curve of the microbial nomogram.

**Figure S2**: Grouping of the mRNA dataset. (**A**) Distribution of risk scores for patients in the high and low risk groups in the training dataset. (**B**) The heatmap of the expression of 4 mRNA. (**C**) Distribution of survival for patients in the high and low risk groups. (**D**) The net benefit of the indicators. (**E**) Calibration curve of the mRNA nomogram.
